# Faces in scenes attract rapid saccades

**DOI:** 10.1167/jov.23.8.11

**Published:** 2023-08-08

**Authors:** Petra Borovska, Benjamin de Haas

**Affiliations:** 1 Experimental Psychology, Justus Liebig University, Giessen, Germany; 2 Experimental Psychology, Justus Liebig University, Giessen, Germany

**Keywords:** face, extrafoveal, complex scenes, fixation duration, saccade peak velocity

## Abstract

During natural vision, the human visual system has to process upcoming eye movements in parallel to currently fixated stimuli. Saccades targeting isolated faces are known to have lower latency and higher velocity, but it is unclear how this generalizes to the natural cycle of saccades and fixations during free-viewing of complex scenes. To which degree can the visual system process high-level features of extrafoveal stimuli when they are embedded in visual clutter and compete with concurrent foveal input? Here, we investigated how free-viewing dynamics vary as a function of an upcoming fixation target while controlling for various low-level factors. We found strong evidence that face- versus inanimate object–directed saccades are preceded by shorter fixations and have higher peak velocity. Interestingly, the boundary conditions for these two effects are dissociated. The effect on fixation duration was limited to face saccades, which were small and followed the trajectory of the preceding one, early in a trial. This is reminiscent of a recently proposed model of perisaccadic retinotopic shifts of attention. The effect on saccadic velocity, however, extended to very large saccades and increased with trial duration. These findings suggest that multiple, independent mechanisms interact to process high-level features of extrafoveal targets and modulate the dynamics of natural vision.

## Introduction

A crucial question in sensory neuroscience is how foveated visual systems combine the processing of upcoming eye movements with that of currently fixated stimuli to manage the alternating flow of fixations and saccades. A vast literature on transsaccadic integration shows that features of an upcoming target can be processed before a saccade is initiated (Herwig & Schneider, 2014; Osterbrink & Herwig, 2021; Wilmott & Michel, 2021). In tasks presenting isolated stimuli, face-directed saccades show lower latency (Broda & de Haas, 2022a; Crouzet, Kirchner, & Thorpe, 2010) and higher velocity (Xu-Wilson, Zee, & Shadmehr, 2009) than those directed to control inanimate objects. However, it is unclear to which degree this translates to gaze dynamics during the natural cycle of saccades and fixations during free-viewing. In natural scenes, the upcoming target typically is embedded in visual clutter, and the programming of a saccade occurs in parallel to the processing of the currently foveated stimulus. Do faces affect gaze dynamics under these conditions in a similar way?

### An effect of faces on peak velocity

It has long been thought that peak velocity forms a stereotypical relationship with saccade amplitude, which is insensitive to changes in stimulus properties (Xu-Wilson et al., 2009). This relationship is referred to as “main sequence” (Bahill, Clark, & Stark, 1975): Peak velocity increases linearly with amplitude, up to a saturation point (Rigas, Komogortsev, & Shadmehr, 2016). Later studies have used saccadic choice paradigms and isolated stimuli to show that this saturation point, as well as the steepness of the linear fit, can differ between observers (Reppert, Lempert, Glimcher, & Shadmehr, 2015) and crucially also be increased for faces as targets (Kauffmann et al., 2019; Xu-Wilson et al., 2009). The study by Xu-Wilson et al. (2009) has shown that saccades to locations expected to show isolated face stimuli, compared to isolated inanimate objects or random pixel noise, had higher velocities and shorter duration, although the effect was relatively small (5.48 dva/s higher for faces on average). A recent study by Yoon, Geary, Ahmed, and Shadmehr (2018) suggests that isolated faces can be understood as items with high reward value, provoking increased vigor (i.e., effort to reach them quickly).

Saccades toward a suddenly appearing stimulus in a saccadic choice task are, however, mostly reactive and may thus differ substantially from voluntary saccade generation during free-viewing (Gremmler & Lappe, 2017; Xu-Wilson et al., 2009). Moreover, natural scene viewing is marked by visual clutter and the concurrent processing of foveal and extrafoveal input. It is unclear whether the velocity advantage for face-directed saccades generalizes to such more natural free-viewing conditions.

### An effect of faces on preceding fixation duration

A predictive model of saccade behavior during free-viewing of naturalistic scenes can be improved by including a shift of attention to the upcoming target location already during the preceding fixation (Schwetlick, Rothkegel, Trukenbrod, & Engbert, 2020). According to this model, this kind of preview contributes to the decision on how long to stay at the currently fixated location. Fixation duration has indeed been shown to be modulated by low-level properties of the upcoming target such as contrast and saturation (Einhäuser, Atzert, & Nuthmann, 2020). However, as discussed in a variety of studies focusing on currently foveated stimuli (Henderson & Pierce, 2008; Kümmerer & Bethge, 2021; Kümmerer, Wallis, Gatys, & Bethge, 2017; Xu, Jiang, Wang, Kankanhalli, & Zhao, 2014), such low-level properties do not fully account for gaze dynamics and high-level, semantic features can improve model performance. One of the most salient types of semantic targets in natural scenes are faces. A number of eye-tracking studies have shown that faces are preferentially targeted (Coutrot & Guyader, 2014; Foulsham, Cheng, Tracy, Henrich, & Kingstone, 2010) and fixated longer than other types of inanimate objects during free-viewing of natural scenes (Guo, Mahmoodi, Robertson, & Young, 2006). Whether faces as targets also modulate the duration of the *preceding* fixation during free-viewing is not entirely clear.

As mentioned, lower saccadic latencies for faces have been found in saccadic choice tasks (Broda & de Haas, 2022b; Crouzet et al., 2010), in which isolated stimuli suddenly appear in opposite hemifields and participants have to saccade to a predefined semantic target category. These tasks use a “gap design” in which the preceding fixation dot disappears just before the onset of target and distractor to minimize its effect on latency and have documented “ultrarapid” saccades with latencies as low as 100 ms toward faces. This is in stark contrast to natural viewing conditions, in which the currently fixated part of a scene and the upcoming target have to be processed in parallel and targets are embedded in the scene and thus visual clutter (Nuthmann, 2017).

Few studies have investigated to which degree lower saccadic latencies in choice tasks generalize to shorter preceding fixations during free-viewing. Cerf, Paxon Frady, and Koch (2009) found that the very first saccade directed toward a scene had lower latency when it was directed toward faces or text rather than cell phones. Similarly, Martin, Davis, Riesenhuber, and Thorpe (2018) recently found that “ultrarapid” saccades generalize to faces superimposed on a scene background. Most important, Mackay, Cerf, and Koch (2012) found that the first few saccades on a complex scene (following the initial one) could be preceded by short fixations and predicted by a salience model, including an explicit face channel. However, fixation durations during scene viewing are known to be shaped by several oculomotor and low-level factors (Tatler & Vincent, 2008), which were not considered in these previous studies. For example, the angle and amplitude of an incoming saccade can predict the magnitude of the following (outgoing) saccade and in turn the duration of the intermittent fixation (Schwetlick et al., 2020; Tatler & Vincent, 2008; Tatler, Brockmole, & Carpenter, 2017). Specifically, short saccades are likely to be followed by saccades in either a similar or the opposite direction, and a fixation between two saccades with similar direction is likely to be of short duration (Schwetlick et al., 2020). Moreover, target size and low-level saliency features such as local luminance contrast at the current and target locations can impact fixation duration (Dick, Ostendorf, Kraft, & Ploner, 2004; Tatler et al., 2017). As of yet, it is unclear how such oculomotor and low-level factors may interact with or confound the effect of faces on fixation durations during free-viewing.

Taken together, previous findings suggest that faces as targets provoke low-latency, high-velocity saccades. However, it is unclear to which degree these effects generalize to free-viewing, especially when controlling for other known factors of oculomotor dynamics. Here, we used a large data set of more than 100 observers free-viewing hundreds of complex scenes, containing close to 50,000 relevant saccadic events (around 40,000 inanimate object–directed and 7,000 face-directed saccades complying with stringent selection criteria). This allowed us to test whether human viewing dynamics are modulated by semantic properties of the upcoming saccade target during the natural cycle of fixations and saccades, taking into account a range of low-level factors known to modulate gaze dynamics.

**Figure 1.**
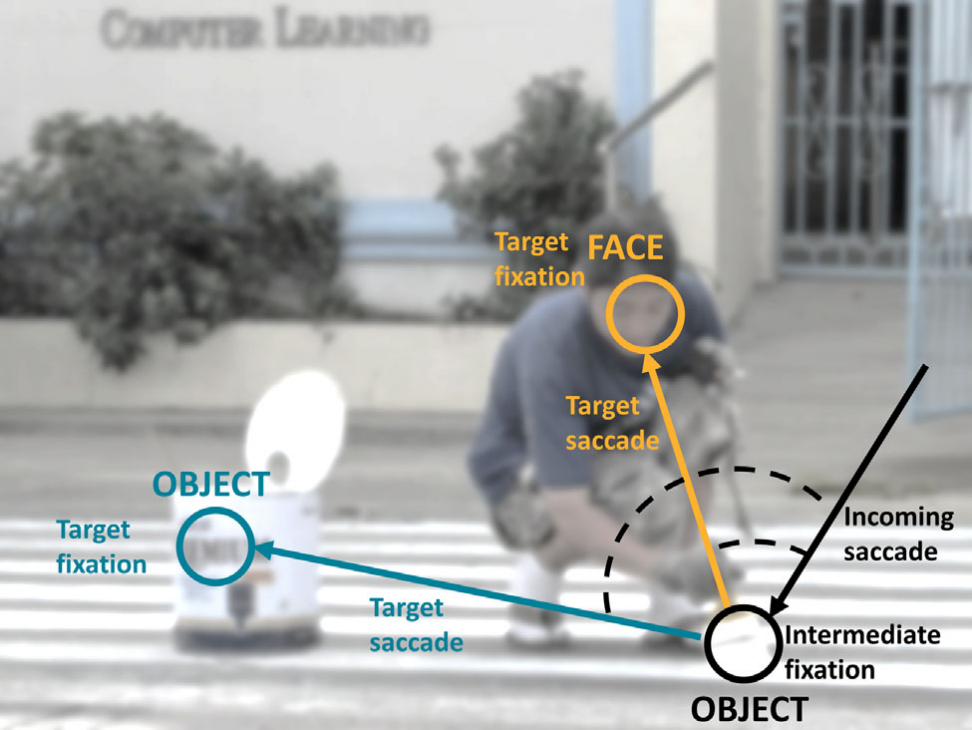
A sequence of incoming saccade, intermediate fixation, target saccade, and target fixation overlaid on an example image. We identified face-related and inanimate object–related saccade as target saccades that landed either on an inner face region (orange example) or an inanimate object (cyan example). Our dependent variables were the peak velocity of the target saccade and the duration of the preceding intermediate fixation. Independent control variables included the amplitude and peak velocity of the incoming saccade and the angle between incoming saccade and target saccade (dashed lines). Note the example image shown has been blurred for illustrative purposes.

Specifically, we compared the peak velocity of face-directed versus inanimate object–directed saccades and the duration of preceding (inanimate object–directed) fixations. We hypothesized that saccades targeting faces (1) have higher peak velocity and (2) are preceded by shorter fixation durations. The size of our data set allowed us to control for a range of potential confounds and modulators occurring under natural viewing conditions that have been reported to affect saccade latency and/or velocity: saccadic amplitude of the incoming and target saccades (cf. Figure 1; Tatler & Vincent, 2008; Xu-Wilson et al., 2009), trial time (Nuthmann, 2017; Tatler et al., 2017), relative angle of incoming and outgoing saccades (Schwetlick et al., 2020), target size (Dick et al., 2004; Guadron, van Opstal, & Goossens, 2022), and low-level salience at the preceding fixation and target locations (Einhäuser et al., 2020; Harel, Koch, & Perona, 2007; Nuthmann, 2017). We controlled for these predictors because we expected that they matter for peak velocity and/or preceding fixation duration based on previous literature. Specifically, previous findings suggest that peak velocity increases with target saccade amplitude (i.e., the main sequence; Bahill et al., 1975), peak velocity decreases across trial time (Unema, Pannasch, Joos, & Velichkovsky, 2005), fixation duration increases with the angle between incoming and target saccades (Schwetlick et al., 2020), and fixation duration increases across trial time (Unema et al., 2005). However, we had no strong expectations how these predictors would interact with the effect of faces and consider the corresponding analyses exploratory.

To foreshadow our results, we found clear evidence that face-directed saccades have higher peak velocities and are preceded by shorter fixation durations. Interestingly, the effect of shorter preceding fixation durations is limited to face-directed saccades with relatively low amplitudes, following inanimate object–directed saccades of similar direction and occurring early in a trial. This may point to an interaction between face-channel and saccade-related retinotopic shifts of attention (Mackay et al., 2012; Schwetlick et al., 2020). At the same time, the effect on saccadic velocity generalizes to large saccades and increases over trial time, suggesting that multiple, dissociable mechanisms process high-level features outside the fovea to modulate gaze dynamics.

## Materials and methods

### Participants

We reanalyzed an existing data set of 103 participants free-viewing 700 complex scenes. The fixations of these participants were previously analyzed and published (Linka & de Haas, 2020). Here, we extracted and analyzed their saccades. Subjects were recruited at Leibniz Institute of Psychology Trier using the PsychLab offline service. We excluded two subjects from the analysis due to missing data files, leaving a sample of *N* = 101 (*M*_age_ = 25.21; *SD* = 5.54; 8 left-handed; 70 females). All participants had normal or corrected-to-normal vision. The study was approved by the local ethics committee and all participants gave informed consent before the experiment. For details, see Linka and de Haas (2020).

### Apparatus

Participants placed their heads in a chin and forehead rest and viewed stimuli at a distance of ∼64 cm at 29.7 × 22.3 degrees visual angle. The experiment was controlled via Psychtoolbox (Kleiner et al., 2007) and MATLAB (MathWorks, Natick, MA, USA). Gaze data were acquired using an EyeLink 1000 Plus eye tracker (SR Research, Ottawa, Canada) at a frequency of 2 kHz.

### Stimuli and procedure

We used annotated stimuli from the Object and Semantic Images and Eye-tracking (OSIE) data set (Xu et al., 2014). The OSIE contains a total of 700 complex everyday scenes and corresponding pixel masks for 5,551 *visual objects* (we refer to *visual objects* as a superordinate category including both face and inanimate object) with binary labeling for 12 semantic attributes (e.g., *Faces*, *Text*, *Touched*). For details, see Xu et al. (2014). Additionally, we used OSIEplus masks and labels (Broda & de Haas, 2022a), which refine the pixel masks for persons into nine categories (e.g., *Inner faces*, *Heads*, *Eyes*). For details, see Broda and de Haas (2022b). Participants freely viewed all 700 images in seven blocks of 100 images each. Each block was preceded by a calibration. Before each image presentation, a self-paced fixation disk appeared, followed by a display of the image for 3 s. All images were presented in the same order across participants.

### Data and availability

Anonymized data and MATLAB code to reproduce the presented findings are freely available at https://osf.io/vj985/.

### Analysis

#### Preprocessing

Saccades and fixations were extracted by using the SR Research saccade detection algorithm and parser with default values of a minimum velocity of 30°/s and a minimum acceleration of 8,000°/s^2^. Gaze coordinates were mapped to image coordinates and removed if they fell outside of the image borders. To exclude fixations and saccades initiated before image onset, fixations and saccades with an onset time < 100 ms trial time were disregarded, which amounted to 9% of saccades and fixations exclusion on average (Linka & de Haas, 2020; SR Research, 2022). Additionally, fixations with a duration under 100 ms were excluded (SR Research, 2022). This led to an exclusion of 5% of fixations on average. To prevent erroneous gaze estimation during lid occlusion caused by a blink, saccades occurring 100 ms before or after a blink were also discarded (i.e., 5% of fixations on average were removed). We further removed potential corrective saccades (i.e., 0.3% of saccades on average were removed). Corrective saccades were defined as saccades that were smaller than 30% of the preceding saccade and had an angle deviation less than 20 degrees (same-directed) or more than 160 degrees (opposite-directed) to that previous saccade. We also disregarded saccades and fixations with a duration > 1,000 ms (Nuthmann, 2017) or peak velocity > 1,000 deg/s. That led to an exclusion of 0.2% of fixations and saccades on average.

#### Event detection

We identified events of interest for each trial and each participant as intermediate fixations that were preceded and followed by saccades, which we refer to as incoming and target saccade, respectively. This process necessarily excluded the last fixations of the trial and the first saccade of the trial. To label fixations and saccades as falling on a given *visual object*, we used the OSIE pixel masks (see above). We used the additional OSIEplus pixel masks (Broda & de Haas, 2022a) to identify fixations on the *inner face* region of a depicted person (thus excluding, e.g., fixations on the back of the head). A fixation was assigned the label(s) of a given pixel mask if a radius of ∼0.5 degrees visual angle around the nominal fixation center overlapped with the mask (i.e., the approximate area of foveation). We additionally required saccades to have start and landing points on different *visual objects* (Linka & de Haas, 2021). Intermediate fixations had to be on inanimate objects and target fixations on inanimate objects (inanimate object–directed saccades) or the inner region of a human face (face-directed saccades). We also excluded all animal-related saccades (see Supplementary Table S10 for details on frequency of faces, animals, and inanimate objects). This resulted in 6,809 valid face-directed and 42,072 valid inanimate object–directed target saccades across participants and images. Note there could be multiple valid event series for a given observer and image. Figure 1 shows a valid event series, consisting of incoming saccade–intermediate fixation–inanimate object or face-directed target saccade–target fixation.

#### Parameters of interest

To test the potential effect of semantic target category (face vs. inanimate object) on saccade latency and velocity, we tested whether the duration of intermediate fixations (in ms) and the peak velocity of target saccades (in deg/s) varied as a function of target. To test potential interactions and control for potential confounds, we considered several additional independent variables that have been reported to affect saccade latency and/or velocity: amplitude of the target saccade in degrees visual angle (dva) (Tatler & Vincent, 2008; Xu-Wilson et al., 2009), absolute angle (Schwetlick et al., 2020) of the target saccade relative to the incoming saccade in degrees (deg) (Figure 1; with 0 denoting a continuation and 180 a reversal), onset time of the target saccade relative to image onset in ms (i.e., time in trial; Nuthmann, 2017; Tatler et al., 2017), target size (i.e., area of the corresponding pixel mask, expressed as percentage of image area; Dick et al., 2004; Guadron et al., 2022) (see Supplementary Figure S6 for details on size distribution), and graph-based visual saliency (GBVS) (Einhäuser et al., 2020; Harel et al., 2007; Nuthmann, 2017) at the intermediate and target fixation locations (i.e., sum of pixel saliency values in a radius of ∼0.5 dva around the fixation center).

### Statistical analysis

To compare the peak velocity of target saccades landing on faces and inanimate objects, as well as the duration of preceding intermediate fixations, we extracted all relevant events for participants (*N* = 101) and trials (*N* = 700). Statistical tests were conducted in MATLAB R2020b (MathWorks) using the *ttest*, *anovan*, and *fitlme* functions.

We used separate linear mixed-effects models to test for an effect of face versus inanimate object (semantic target category) on target saccade peak velocity and intermediate fixation duration. We used dummy coding for semantic target category, with faces coded as 1 and the reference category of inanimate objects coded as 0. In addition to semantic target category, we included seven further predictors to control for potential confounds (see above): (1) target saccade amplitude, (2) incoming saccade amplitude, (3) size of target stimuli, (4) time from onset of the trial, (5) angle of the target to incoming amplitude, (6) GBVS of intermediate fixation, and (7) GBVS of target fixation. All continuous predictor variables were *z*-scored. The dependent variable peak velocity was *z*-scored and the dependent variable fixation duration was *z*-scored and log-transformed due to the right-skewness of the underlying distribution. We used three random factors in both models: *subject* (101 levels), *image* (591 levels), and *visual object* (2,857 levels). The *images* were crossed with *subjects*, and the *visual objects* were nested in *images* (see Supplementary Table S1 for details). We estimated both an intercept and a slope for *subject* and *image* but not for *visual object* as it was either a face or an inanimate object. We selected the best-fitting model specification based on differences in Akaike's information criterion (AIC), considering both main and random effects. To do this, we iteratively removed one fixed predictor at a time from the model and compared all candidate models to the one with minimal AIC:

$$\begin{array}{c} \Delta_{i}=\;AIC_{i}-\;AIC_{min} \end{array}$$

where *AIC_min_* is the AIC of the model with the lowest AIC among all candidate models, *AIC_i_* is the AIC of the *i*th other candidate model, and Δ_*i*_ designates the difference between their AICs (Burnham & Anderson, 2002). Both models showed the lowest AIC for the full model with all predictors included. If available, we selected the most simple model performing on par with the full model according to AIC (i.e., Δ_*i*_ < 2; Burnham & Anderson, 2002). This was the case only for the fixation duration model, including a random by-*subject* intercept and slope, random by-*image* intercept, and random by-*visual object* intercept (Supplementary Table S2 & Supplementary Table S3). For peak velocity, we selected the full model, including a random by-*subject* slope and intercept, random by-*image* slope and intercept, and random by-*visual object* intercept (Supplementary Table S2 & Supplementary Table S3).

We also estimated covariance parameters for random effects (Supplementary Table S3) and conducted model diagnostics, visually inspecting residual plots (Supplementary Figure S1 & Supplementary Figure S2). The full table of linear mixed-effects model (LMM) results including AIC comparisons is reported in Supplementary Tables S2 to S7.

Furthermore, we ran seven two-way analyses of variance (ANOVAs) for each of the dependent variables of interest (target saccade peak velocity and intermediate fixation duration). Each ANOVA tested a potential interaction effect between semantic target category (inanimate object or face) and one control variable. Specifically, the control variables tested in the seven ANOVAs were (1) the angle between incoming and target saccade, distributed across 15 bins of 12 degrees each; (2) target saccade amplitude, distributed across 14 bins of 1 dva each; (3) target saccade onset time, distributed across 13 bins of 200 ms; (4) target size, distributed across 10 bins of 10% each; (5) GBVS of intermediate fixation, distributed across 10 bins of 40 arbitrary salience units (a.u.) each; (6) GBVS of target fixation, distributed across 40 a.u. each; and (7) incoming saccade amplitude, distributed across 14 bins of 1 dva each. We expected three of these predictors to be of particular importance: the angle between incoming and target saccade, the target saccade amplitude, and target saccade onset time. We show the corresponding ANOVA results in the main text. The full list of ANOVA results is reported in the Supplementary Table S8. For each ANOVA, we ran separate post hoc paired *t*-tests. The significance level of these *t*-tests was determined at a family-wise error rate of α = 0.05 using the Holm–Bonferroni method to correct for multiple testing (asterisks in plots denote significance surviving this correction).

As a post hoc control analysis, we used linear mixed-effects models to test for an effect of time from trial onset and the angle between incoming and target saccades on the amplitude of target saccades. This model also included semantic target category as a control predictor for specific effects of faces and inanimate objects as targets on saccadic amplitude. We controlled for these effects to further explore an initial finding of slower saccades toward the end of the trial (Supplementary Figure S5 & Supplementary Table S9).

Finally, we conducted a control analysis to test whether the effect of faster saccades toward faces and shorter preceding fixation durations is driven by animacy and extends to human bodies (Yun, Peng, Samaras, Zelinsky, & Berg, 2013). We contrasted saccades landing on human bodies (without faces) versus inanimate objects. This resulted in 5,594 valid body-related and 42,072 inanimate object–related target saccades. Again, we used separate linear mixed-effects models to test for an effect of body versus inanimate object on target saccade peak velocity and intermediate fixation durations. Model specifications were identical to the main analysis contrasting faces and inanimate objects. We did not find evidence supporting an effect of animacy and therefore do not report follow-up ANOVAs and *t*-tests here and instead refer interested readers to our OSF repository (osf.io/vj985/).

## Results

### Events of interest

We used data from 101 participants free-viewing 700 complex everyday scenes for 3 s each. We identified events of interest for each trial and participant as an intermediate fixation landing on any inanimate object, followed by a saccade targeting either a face or another inanimate object (Figure 1). Pooled across participants and trials, we found 6,809 such events targeting a face and 42,072 targeting an inanimate object. Figure 1 shows a valid event series, consisting of incoming saccade–intermediate fixation–inanimate object or face-directed target saccade–target fixation.

### Main hypotheses

We tested two main hypotheses regarding the influence of an upcoming face or inanimate object target on free-viewing gaze behavior: (1) Peak velocity will be higher for a saccade targeting faces versus inanimate objects, and (2) the duration of an intermediate inanimate object fixation will be shorter when the following saccade target is a face versus inanimate object. To test simple main effects of semantic target category (face vs. inanimate object), we used linear mixed-effects models for each measure. To control for potential confounds (see Introduction), we included seven additional predictors: (1) target saccade amplitude, (2) incoming saccade amplitude, (3) size of target stimuli, (4) time from onset of the trial, (5) angle of the target to incoming amplitude, (6) GBVS at the intermediate fixation, and (7) GBVS at the target fixation. To test potential modulatory effects of these low-level factors, we additionally ran two-way ANOVAs, testing potential interactions between semantic target category and one control variable at a time.

### Saccade peak velocity

The average peak velocity of saccades targeting a face (*N* = 6,809, *M* = 369.48, *SD* = 104.28, *SE* = 1.26) was indeed higher than that of saccades targeting inanimate objects (*N* = 42,072, *M* = 328.99, *SD* = 112.24, *SE* = 0.54).

#### Linear mixed-effect model of semantic target category and control predictors

**Figure 2.**
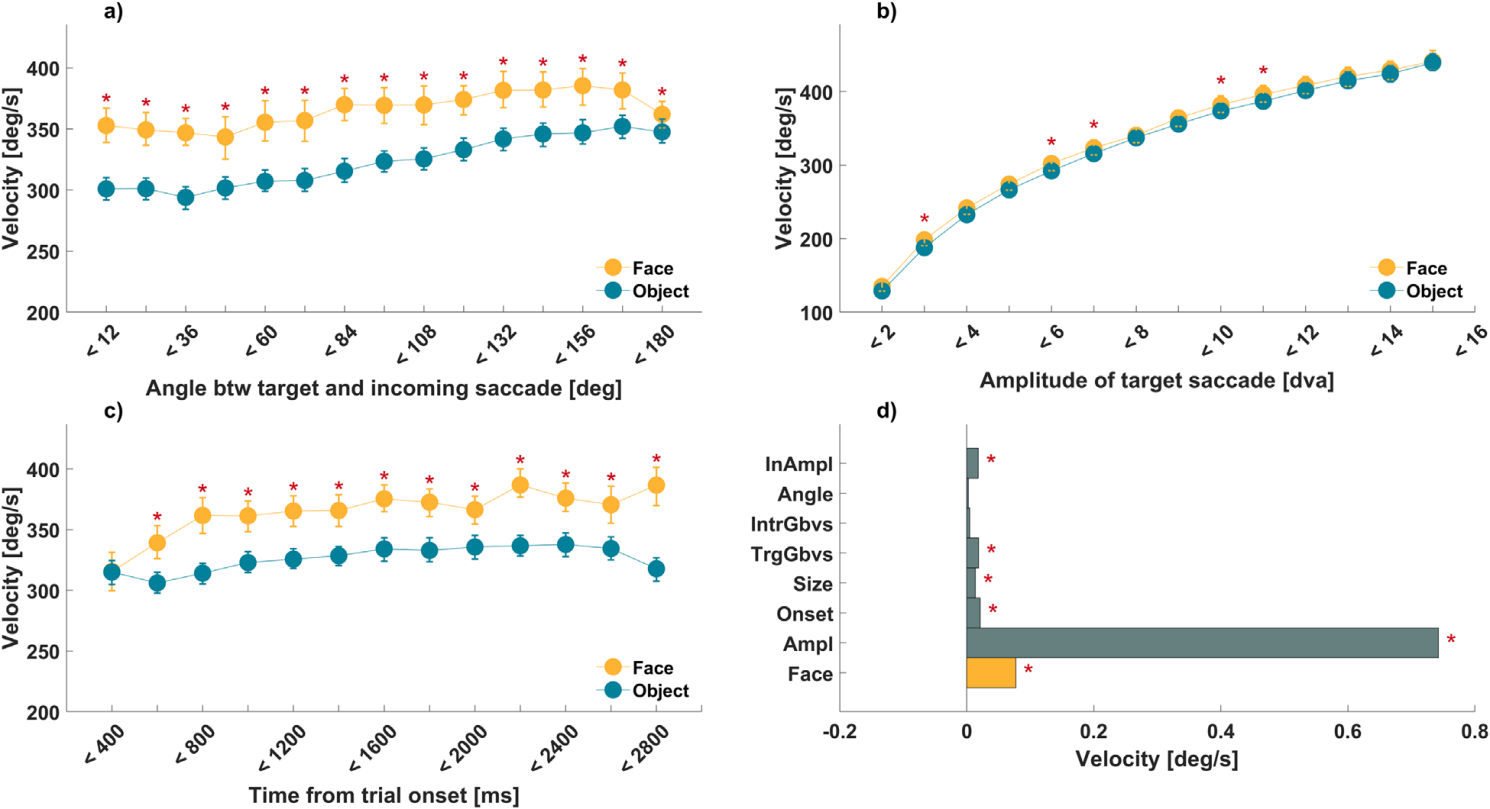
Peak velocity. (**a**–**c**) Peak velocity of target saccades landing on faces and inanimate objects in cyan and yellow, as shown in the inset. Red asterisks mark Bonferroni-corrected significance of paired *t*-test and error bars represent bootstrapped 95% confidence interval (1,000 resamples). (**a**) Peak velocity as a function of absolute deviation of saccade angles between target and incoming saccade. The increase in angle represents an increase from same-directed saccades (<12 degrees) to opposite-directed saccades (<180 degrees). (**b**) Peak velocity as a function of target saccade amplitude (in dva), showing the main sequence. (**c**) Peak velocity as a function of time from onset (ms) within a trial. (**d**) Standardized, fitted predictor weights of a linear mixed-effects model of peak velocity with simple main effects of semantic target category (Face; shown in yellow bar), target amplitude (Ampl), time from trial onset (Onset), size of target stimuli (Size), low-level salience at target (TrgGbvs) and intermediate (IntrGbvs) fixation, absolute deviation of saccade angle between incoming and target saccade (Angle), and amplitude (InAmpl) of the incoming saccade. Red asterisks mark statistically significant beta coefficients. Note that all continuous variables were *z*-scored and thus the corresponding beta values indicate effects in standard deviation units.

To test the statistical significance of this effect and control for potential confounds, we implemented a linear mixed-effect model (for full results, see Supplementary Table S2 & Supplementary Table S3). This confirmed strong evidence for an effect of semantic target category (*b* = 0.07, *SE* = 0.02, *t*(48,872) = 4.33, *p* < 0.001) (Figure 2d), indicating a higher peak velocity for saccades targeting faces versus inanimate objects, even when other relevant predictors were held constant. The model also confirmed the expected strong effect of target saccade amplitude (*b* = 0.74, *SE* = 0.003, *t*(48,872) = 238.72, *p* < 0.001), that is, the main sequence (Bahill et al., 1975). Expressed in standardized weights, the effect of semantic target category amounted to about 10% of that observed for target saccade amplitudes. Additional significant but smaller effects on peak velocity included trial time of saccade onset (*b* = 0.02, *SE* = 0.002, *t*(48,872) = 8.93, *p* < 0.001; indicating saccadic velocity increases as trial time progresses), target size (*b* = 0.01, *SE* = 0.005, *t*(48,872) = 2.52, *p* < 0.05; indicating saccadic velocity increased as the size of the target increased), low-level saliency of the target (*b* = 0.02, *SE* = 0.003, *t*(48,872) = 5.28, *p* < 0.001; indicating higher velocity saccades toward targets with higher low-level saliency), and amplitude of the incoming saccade (*b* = 0.02, *SE* = 0.002, *t*(48,872) = 7.43, *p* < 0.001; indicating higher velocity target saccades for larger incoming saccades). Finally, the effects of low-level saliency at the intermediate location (*b* = 0.004, *SE* = 0.002, *t*(48,872) = 1.55, *p* = 0.12) and angle between incoming and target saccade (*b* = 0.002, *SE* = 0.002, *t*(48,872) = 0.98, *p* = 0.32) were small and not statistically significant. For details, see Supplementary Table S2 and Supplementary Table S3.

#### Targeted two-way ANOVAs

We additionally ran two-way ANOVAs to test potential interactions between semantic target category and the remaining predictors. The first of these models tested the simple main effects of target category, *F*(1, 48,880) = 777.4, *p* < 0.05, η^2^ = 0.015, and deviation of the target saccade angle from that of the incoming saccade, *F*(14, 48,880) = 38.35, *p* < 0.05, η^2^ = 0.011. Unlike in the LMM results, the simple main effect of angle was significant. Holm–Bonferroni corrected post hoc paired *t*-tests showed faster saccades toward faces versus inanimate objects across all relative angles (Figure 2a). However, there also was a significant interaction, *F*(14, 48,880) = 4.48, *p* < 0.05, η^2^ = 0.001, indicating a higher velocity advantage for face-targeting saccades with a similar angle to the incoming one.

The second two-way ANOVA tested and confirmed significant simple main effects of target category, *F*(1, 45,048) = 53.27, *p* < 0.05, η^2^ = 0.001, and target saccade amplitude, *F*(13, 45,048) = 1,703.03, *p* < 0.05, η^2^ = 0.33, but no significant interaction between the two, *F*(13, 45,048) = 0.77, *p* = 0.68, η^2^ = 0.0002. As shown in Figure 2b and Supplementary Table S8, Holm–Bonferroni corrected post hoc *t*-tests indicated significantly faster saccades toward faces versus inanimate objects for most amplitude bins, up to 12 dva (with an average advantage of 7.18 deg/s).

The third two-way ANOVA tested and confirmed significant simple main effects of target category, *F*(1, 48,875) = 696.88, *p* < 0.05, η^2^ = 0.01, and trial onset time, *F*(12, 48,875) = 21.37, *p* < 0.05, η^2^ = 0.005. Figure 2c shows significant Holm–Bonferroni corrected post hoc *t*-tests for almost all onset bins, except very early saccades under 400 ms. A significant interaction, *F*(12, 48,875) = 5.75, *p* < 0.05, η^2^ = 0.001, pointed to a general increase of the velocity advantage for face-directed saccades over trial time.

#### Remaining predictors

For completeness, we also ran additional ANOVAs for all remaining predictors (cf. Supplementary Results, Table S8). We found significant interactions between target category and each of the following factors: the target size (*F*(9, 48,070) = 24.01, *p* < 0.05, η^2^ = 0.004; indicating a higher velocity advantage for smaller faces), low-level saliency of the intermediate fixation location (*F*(9, 13,727) = 2.39, *p* < 0.05, η^2^ = 0.001; indicating a more pronounced velocity advantage for faces when low-level saliency at the intermediate location was high), and amplitude of the incoming saccade (*F*(13, 41,496) = 2.41, *p* < 0.05, η^2^ = 0.0008; indicating a higher velocity advantage for faces for higher amplitudes of the incoming saccade).

#### Interim summary, Part 1

Taken together, these results showed higher velocities for face- compared to inanimate object–directed saccades. This effect was substantial (about 10% of that of the main sequence) and robust to controlling for a range of other factors it interacted with. These interactions point to a velocity effect of faces for saccades of all amplitudes, increasing over trial time, being largest for saccades continuing a large incoming saccade in a straight line and when low-level salience at the intermediate fixation location is high.

### Fixation duration

We compared the fixation duration at intermediate fixation locations in milliseconds when the following saccade landed on faces (*N* = 6,809, *M* = 224.38, *SD* = 106.4 , *SE* = 1.29) versus inanimate objects (*N* = 42,072, *M* = 224.70, *SD* = 101.77, *SE* = 0.49). The simple means of fixation durations for both types of fixations were not statistically different.

#### Linear mixed-effect model of semantic target category and control predictors

**Figure 3.**
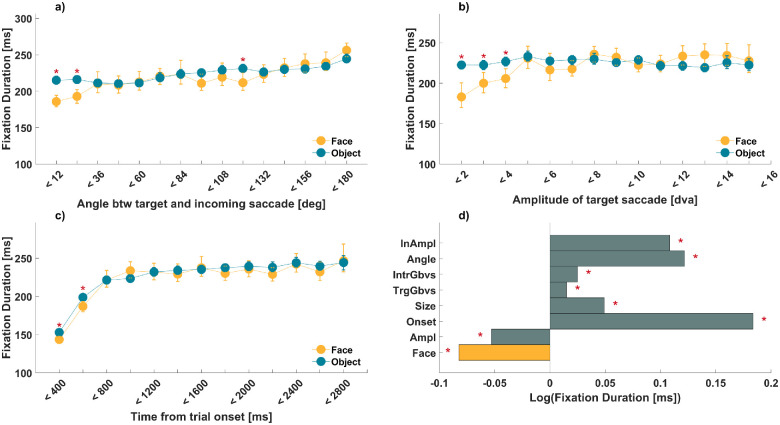
Measures of fixation duration. (**a**–**c**) Intermediate fixation duration (ms) followed by saccade landing on faces and inanimate objects in yellow and cyan, as shown in the inset. Red asterisks mark Bonferroni-corrected significance of paired *t*-test and error bars represent bootstrapped 95% confidence interval (1,000 resamples). (**a**) Intermediate fixation duration as a function of absolute deviation of saccade angles between target and incoming saccade. The increase in angle represents an increase from same-directed saccades (<12 degrees) to opposite-directed saccades (<180 degrees). (**b**) Intermediate fixation duration as a function of target saccade amplitude (in dva). (**c**) Intermediate fixation duration as a function of time from onset (ms) within a trial. (**d**) Standardized, fitted predictor weights of a linear mixed-effects model of intermediate fixation duration with simple main effects of semantic target category (Face; shown in yellow bar), target amplitude (Ampl), time from trial onset (Onset), size of target stimuli (Size), low-level salience of target (TrgGbvs) and intermediate (IntrGbvs) fixation, absolute deviation of saccade angle between incoming and target saccade (Angle), and amplitude of the incoming saccade (InAmpl). Red asterisks mark statistically significant beta coefficients. Note that all continuous variables were *z*-scored and thus the corresponding beta values indicate effects in standard deviation units.

We implemented a similar linear mixed-effect model as for peak velocity to test the statistical significance of the effect of face targets on intermediate fixation durations when other predictors were held constant. We observed a significant, negative effect of semantic target category (*b* = –0.08, *SE* = 0.02, *t*(48,872) = –4.09, *p* < 0.001; Figure 3d), indicating shorter intermediate fixation for face-directed target saccades. A range of further predictors had significant and sometimes strong effects on the duration of intermediate fixations: time from trial onset (*b* = 0.18, *SE* = 0.004, *t*(48,872) = 40.96, *p* < 0.001; indicating longer intermediate fixation durations as trial time progress), absolute angle between target and incoming saccade (*b* = 0.12, *SE* = 0.005, *t*(48,872) = 26.09, *p* < 0.001; indicating longer intermediate fixation durations preceding saccades that reversed direction), amplitude of the incoming saccade (*b* = 0.11, *SE* = 0.005, *t*(48,872) = 23.38, *p* < 0.001; indicating longer intermediate fixation durations for larger incoming saccades), amplitude of target saccade (*b* = –0.05, *SE* = 0.005, *t*(48,872) = –9.53, *p* < 0.001; indicating longer intermediate fixation durations for smaller target saccades), size of the target (*b* = 0.04, *SE* = 0.007, *t*(48,872) = 6.76, *p* < 0.001; indicating longer intermediate fixation durations when the following saccade landed on a larger target), low-level salience at the intermediate fixation location (*b* = 0.02, *SE* = 0.005, *t*(48,440) = 4.62, *p* < 0.001; indicating longer intermediate fixation durations for higher low-level saliency of the currently fixated inanimate object), and low-level salience of the target (*b* = 0.01, *SE* = 0.005, *t*(48,872) = 4.62, *p* < 0.001; indicating longer intermediate fixation durations for higher low-level saliency of the target). For details, see Supplementary Table S2 and Supplementary Table S3.

#### Targeted two-way ANOVAs

Similar as for peak velocity, we ran additional two-way ANOVAs to test potential interactions between semantic target category and other predictors in modulating intermediate fixation duration. The first, two-way ANOVA revealed significant simple main effects of semantic target category, *F*(1, 48,880) = 10.12, *p* < 0.05, η^2^ = 0.0002, and absolute deviation angle between the incoming and target saccades, *F*(14, 48,880) = 33.85, *p* < 0.05, η^2^ = 0.009. A significant interaction of semantic category and angle, *F*(14, 48,880) = 4.73, *p* < 0.05, η^2^ = 0.001, indicated the shortest fixation durations preceded face-targeting saccades in the same direction as the incoming saccade, but this effect of faces was diminished or even reversed for saccades going in the opposite direction. This was confirmed by post hoc Holm–Bonferroni corrected paired *t*-test showing shorter fixation durations for faces in same-directed saccades, *t*(97) = −7.1, *p* < 0.05, *t*(93) = −4.69, *p* < 0.05, and for saccades in almost opposite directions (angle of 120 degrees), *t*(96) = −3.3, *p* < 0.05 (Figure 3e).

A second two-way ANOVA showed a significant simple main effect of semantic target category, *F*(1, 45,048) = 4.83, *p* < 0.05, η^2^ = 0.0001, and target saccade amplitude, *F*(13, 45,048) = 3.81, *p* < 0.05, η^2^ = 0.001. A significant interaction, *F*(13, 45,048) = 4.23, *p* < 0.05, η^2^ = 0.001, indicated that shorter fixation durations precede face-directed saccades of small amplitudes. This was confirmed by post hoc Holm–Bonferroni corrected paired *t*-tests, which only showed significant face effects for the shortest amplitudes for 2 dva, *t*(64) = −4.09, *p* < 0.05; 3 dva, *t*(94) = −3.18, *p* < 0.05; and 4 dva, *t*(91) = −3.71, *p* < 0.05 (Figure 3f).

A third two-way ANOVA revealed significant simple main effects of semantic category, *F*(1, 48,875) = 5.91, *p* < 0.05, η^2^ = 0.0001, and onset time, *F*(12, 48,875) = 91.7, *p* < 0.05, η^2^ = 0.02. The interaction between these factors missed statistical significance, *F*(12, 48,875) = 1.73, *p* = 0.053, η^2^ = 0.0004. Nevertheless, shorter fixation duration preceding face-directed saccades seemed limited to early trial times up to 600 ms, as indicated by Holm–Bonferroni corrected paired *t*-test, *t*(95) = −4.35, *p* < 0.05, *t*(91) = −3.3, *p* < 0.05 (cf. Figure 3g).

#### Remaining predictors

For completeness, we also ran additional ANOVAs for all remaining predictors (cf. Supplementary Results, Table S8). Although some of the interactions regarding the effect of faces as targets were significant, these effects appeared unsystematic.

#### Interim summary, Part 2

Taken together, when controlling for a range of potential confounds, intermediate fixation durations depended on the following saccade target. Fixations preceding face-directed saccades were shorter than those preceding saccades directed to inanimate objects. This effect was small to moderate compared to other factors, such as trial time, and interacted with two other predictors: Intermediate fixations are shortest before small face-directed saccades at an angle continuing the incoming saccade. Additionally, the effect appears limited to the first 600 ms of a trial.

#### Fixation duration and peak velocity toward bodies

To probe whether rapid saccades to faces are due to a general animacy effect (Yun et al., 2013), we repeated the main analyses for saccades targeting bodies instead of faces. We implemented linear mixed-effect models to test for an effect of bodies versus inanimate objects as targets on the peak velocity of target saccades and on the duration of preceding intermediate fixations.

**Figure 4.**
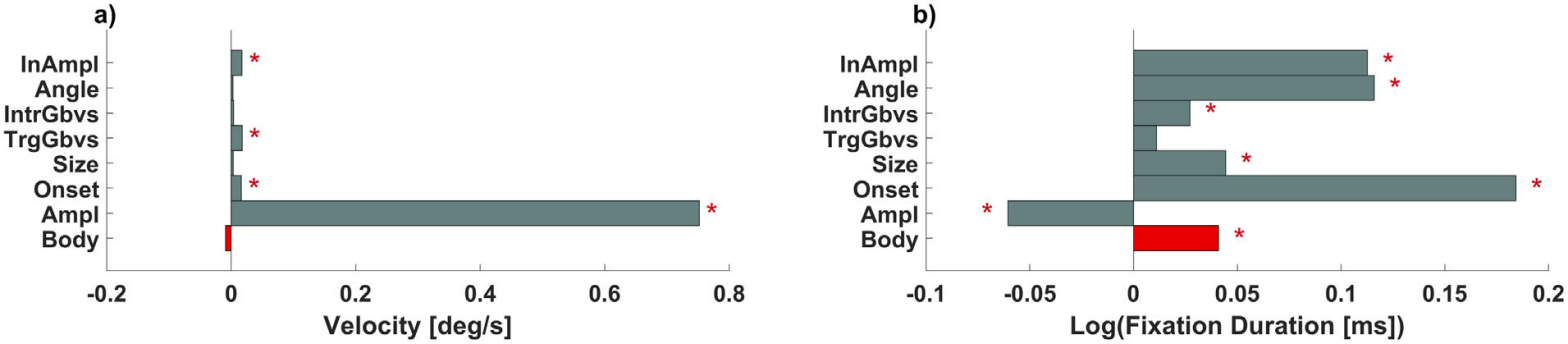
Linear mixed-effects models fitting standardized velocity and log fixation duration. Standardized, fitted predictor weights of a linear mixed-effects model of peak velocity (**a**) and intermediate fixation duration (**b**) with simple main effects of target category (Body; shown in red bar), target amplitude (Ampl), time from trial onset (Onset), size of target stimuli (Size), low-level salience of target (TrgGbvs) and intermediate (IntrGbvs) fixation, absolute deviation of saccade angle between incoming and target saccade (Angle), and amplitude of the incoming saccade (InAmpl). Red asterisks mark statistically significant beta coefficients. Note that all continuous variables were *z*-scored and thus the corresponding beta values indicate effects in standard deviation units.

We observed a nonsignificant, negative effect of target category on peak velocity (*b* = −0.008, *SE* = 0.01, *t*(47,657) = −0.58, *p* = 0.55; Figure 4a), indicating no evidence for body-targeting saccades to be faster than saccades targeting inanimate objects. As in the main analysis, there was a range of further significant predictors: the target saccade amplitude (*b* = 0.75 *SE* = 0.003, *t*(47,657) = 245.73, *p* < 0.001; indicating increasing saccade speed with amplitude), time from trial onset (*b* = 0.01, *SE* = 0.002, *t*(47,657) = 6.91, *p* < 0.001; indicating saccadic velocity increases as trial time progresses), low-level salience of the target (*b* = 0.01, *SE* = 0.003, *t*(47,657) = 5.18, *p* < 0.001; indicating higher velocity saccades toward targets with higher low-level saliency), and amplitude of the incoming saccade (*b* = 0.01, *SE* = 0.002, *t*(47,657) = 7.08, *p* < 0.001; indicating higher velocity target saccades for larger incoming saccades).

Similarly, a linear mixed-effects model showed a reversed effect on preceding intermediate fixation durations, with fixations preceding body-directed saccades lasting longer than saccades preceding inanimate object–directed saccades (*b* = 0.04, *SE* = 0.01, *t*(47,657) = 2.08, *p* < 0.05). Further significant predictors showed a similar profile to those in the main analysis: time from trial onset (*b* = 0.18, *SE* = 0.004, *t*(47,657) = 40.63, *p* < 0.001; indicating longer intermediate fixation durations as trial time progress), absolute angle between target and incoming saccade (*b* = 0.11, *SE* = 0.004, *t*(47,657) = 24.63, *p* < 0.001; indicating longer intermediate fixation durations preceding saccades that reversed direction), amplitude of the incoming saccade (*b* = 0.11, *SE* = 0.004, *t*(47,657) = 24.04, *p* < 0.001; indicating longer intermediate fixation durations for larger incoming saccades), amplitude of target saccade (*b* = −0.06, *SE* = 0.005, *t*(47,657) = −10.92, *p* < 0.001; indicating longer intermediate fixation durations for smaller target saccades), size of the target (*b* = 0.04, *SE* = 0.007, *t*(47,657) = 6.31, *p* < 0.001; indicating longer intermediate fixation durations when the following saccade landed on a larger target), and low-level salience at the intermediate fixation location (*b* = 0.02, *SE* = 0.005, *t*(47,657) = 5.03, *p* < 0.001; indicating longer intermediate fixation durations for higher low-level saliency of the currently fixated inanimate object).

#### Interim summary, Part 3

We found no evidence for rapid saccades toward bodies. Body-directed saccades were not significantly different from saccades toward inanimate objects with respect to peak velocity (Figure 4a). Regarding saccadic latency, fixations preceding body-directed saccades lasted *longer* than those preceding saccades toward inanimate objects (Figure 4b).

## Discussion

### Shorter fixation and higher peak velocity as evidence of extrafoveal processing of high-level features

In the present study, we investigated whether high-level properties of extrafoveal *visual objects* in a complex scene can modulate free-viewing dynamics *before* they are fixated. We found strong evidence that face- versus inanimate object–directed saccades are preceded by shorter fixations and have higher peak velocity. These results are in line with previous findings on the latency and velocity advantage for saccades directed to isolated faces (Broda, Haddad, & de Haas, 2022; Crouzet et al., 2010; Kauffmann, Khazaz, Peyrin, & Guyader, 2021; Reppert et al., 2015; Xu-Wilson et al., 2009; Yoon, Jaleel, Ahmed, & Shadmehr, 2020) and show it extends to free-viewing complex scenes, which is marked by visual clutter and the concurrent processing of foveal and extrafoveal input. The concurrent processing of high-level features at both currently foveated and target locations is matching findings from the transsaccadic literature showing that features of the upcoming target can be processed before the saccade is initiated (Herwig & Schneider, 2014; Osterbrink & Herwig, 2021; Wilmott & Michel, 2021). It also matches the notion that peripheral vision is enhanced by foveal feedback, aiding object recognition (Stewart, Valsecchi, & Schütz, 2020). The rich data set we used allowed us to control for a range of potential confounds and moderators, revealing that the effect of faces on free-viewing dynamics is modulated by target eccentricity, the trajectory of consecutive saccades, and the time from trial onset. Taken together, our results provide strong evidence for the extrafoveal processing of high-level features in natural vision and reveal related moderators that point to potential underlying mechanisms.

### Faces in scenes

Human gaze behavior is systematic, and much research has been devoted to predicting where humans look in a scene when and for how long. Two major approaches (Henderson, 2011) focus on (1) features of the scene (e.g., Itti & Koch, 2000) or (2) top-down control (e.g., Yarbus, 1965). More recent efforts are trying to combine both and emphasize high-level features of scenes (e.g., Einhäuser, Spain, & Perona, 2008; Xu et al., 2014) and faces in particular (Cerf, Harel, Einhäuser, & Koch, 2008; Kümmerer, Wallis, & Bethge, 2016; Xu et al., 2014). In laboratory paradigms, a number of studies have shown that faces are preferentially targeted (Coutrot & Guyader, 2014; Foulsham et al., 2010) and longer fixated (Guo et al., 2006), and saccades toward them tend to be faster (Xu-Wilson et al., 2009). Faces are deemed high-value targets by several studies (Xu-Wilson et al., 2009; Yoon et al., 2018), and adding a face channel to low-level saliency models significantly improves gaze prediction (Cerf et al., 2008). Our results show that faces, but not bodies or inanimate objects, attract rapid saccades during scene viewing. This corroborates the special role of faces for human gaze behavior (see Results; Figure 4).

Studies of occipitotemporal face processing find a strong central visual field bias (e.g., Levy, Hasson, Avidan, Hendler, & Malach, 2001). This is interesting in light of our findings, which show a strong central bias for the effect of faces on saccadic latency (i.e., preceding fixation durations) but not for that on saccadic velocity, which generalized to the periphery (also see below).

### The effect of faces on saccadic velocity

Face-directed saccades had higher peak velocity compared to inanimate object–directed saccades, and this effect of target held even when controlling for various other factors known to modulate saccade velocity. The main factor determining peak velocity is amplitude, resulting in the main sequence relationship (Bahill et al., 1975; Reppert et al., 2015). Interestingly, the effect of faces on peak velocity was constant throughout the amplitude spectrum, even for large saccades.

Velocity also increased with trial time and did so more strongly for face- versus inanimate object–directed saccades. This is in contrast to the general scene-viewing tendency of shorter saccade amplitudes and thus slower saccades toward the end of viewing time (Unema et al., 2005). This apparent discrepancy can be explained by the fact that we limited our analysis to saccade events moving from one *visual object* to another, and many of the small, slow saccades toward the end of a trial inspect successive details within a given *visual object* (focal vs. ambient mode; Nuthmann, 2017; Trevarthen, 1968). Indeed, our data show that the magnitude of saccades for the types of events we selected *increased* over time (see Supplementary Figure S5 & Supplementary Table S9). A possible explanation is that saccades can move more easily between *visual objects* at a greater distance toward the end of a trial, because the target or close-by regions have been visited previously.

The velocity advantage for face-directed saccades was also somewhat larger when incoming and target saccades followed the same trajectory. Importantly, however, the velocity of face-directed saccades is higher than that of inanimate object–directed saccades, independently of low-level salience. This is in line with the importance of semantic features for fixation locations in complex scenes (Itti & Koch, 2000; Mackay et al., 2012; Xu et al., 2014) and extends their importance to the corresponding saccade dynamics.

Previous studies found higher velocities for speeded saccades to isolated faces (Xu-Wilson et al., 2009). This was interpreted to reflect a high intrinsic reward value of faces, as targets associated with reward, such as food (Takikawa, Kawagoe, Itoh, Nakahara, & Hikosaka, 2002) or monetary profit (Chen, Chen, Zhou, & Mustain, 2014), that have been shown to elicit saccades with an increased velocity profile as well. Our results suggest that this effect holds for natural vision too (i.e., the free-viewing of complex scenes), which is marked by visual clutter and the concurrent processing of foveal and extrafoveal input. Free-viewing typically elicits self-paced voluntary as opposed to reactive saccades (Gremmler & Lappe, 2017). Interestingly, the overall peak velocity advantage we observe for faces versus inanimate objects (advantage of 7.18 deg/s) is even larger than that which has been reported in the context of reactive saccades (advantage of 5.48 deg/s; Xu-Wilson et al., 2009). A modulation of viewing dynamics during natural vision appears adaptive. It could help with the time-critical prioritization of conspecifics in visual clutter or of targets in a foraging situation, such as searching for fruit in a canopy.

### The effect of faces on preceding fixation durations

Saccades targeting faces versus inanimate objects were preceded by shorter fixations when controlling for potentially confounding predictors. This is reminiscent of the very low latencies observed for saccades directed to isolated face stimuli (Broda et al., 2022; Crouzet et al., 2010; Martin et al., 2018). Our results show this effect extends to free-viewing, where extrafoveal targets are processed concurrently with currently foveated targets. This seems remarkable, given saccadic choice paradigms typically use a gap design, in order to avoid any concurrent foveal input, including that of a fixation dot.

Importantly, the duration effect we observed here is limited to fixations preceding small saccades following a trajectory similar to the preceding one. This matches the hypothesis of a perisaccadic attentional spotlight shifting in retinotopic coordinates when the saccade is executed (Schwetlick et al., 2020). Schwetlick et al. (2020) recently provided modeling evidence for the decoupling of covert attention and current fixation position in target selection, followed by a brief retinotopic shift of attention in the direction of the saccade, until it is realigned with the current fixation position. Our current results suggest that the resulting pull along the saccadic trajectory is especially pronounced if the shifted postsaccadic window of attention falls on a parafoveal face.

Interestingly, the effect of upcoming face targets on fixation duration seemed limited to saccades occurring within the first 600 ms of a trial, which is in line with previous findings (Mackay et al., 2012) and reminiscent of the notion of ambient versus focal processing (Nuthmann, 2017; Trevarthen, 1968). This co-occurred with a general increase of fixation duration with time from trial onset, which is a well-established finding (Tatler et al., 2017; Unema et al., 2005).

Our findings also suggest that an upcoming face target can only shorten intermediate fixation durations when the face is closer than 4 dva. This may be related to the perisaccadic attentional shifts discussed above, because saccades of a similar direction to the preceding one tend to be small (Schwetlick et al., 2020). It may also point to the involvement of face-selective neurons with a strong central bias in their visual field coverage (see above and below). An important caveat is that most of the faces in the stimulus set we used were rather small (most inner face regions extend < 3 dva). Target size was a positive predictor of preceding fixation durations in our data set, but we cannot rule out that very large faces may shorten saccadic latencies at higher eccentricities. This could be investigated in future studies sampling face eccentricities and sizes more systematically and comprehensively.

Taken together, the effect of face targets on preceding fixation durations appeared more limited than that on peak velocity. Fixation durations were only modulated at the beginning of a trial and for nearby faces, whereas the effect on peak velocity was observed even for the largest saccades and *increased* across trial duration. This suggests that the threshold for an extrafoveal target to modulate the preceding fixation duration may be higher than for modulating saccadic velocity. For instance, the memory of a face at a peripheral location may be sufficient to elicit a saccade with higher velocity, whereas a shortening of the preceding fixation may require the direct parafoveal registering of a face that has not been fixated before. Although speculative at this point, this may also be reflected in the underlying biological mechanisms. The modulation of preceding fixation durations may require the activation of face-sensitive neurons in the ventral stream, which have a strong central bias in their visual field coverage (Gomez, Natu, Jeska, Barnett, & Grill-Spector, 2018; Issa & Dicarlo, 2012; Kay, Weiner, & Grill-Spector, 2015), whereas the modulation of peak velocity may rest on a different (possibly subcortical) face channel with wider visual field coverage.

### Future research and limitations

In terms of future research, even bigger data sets would allow to examine possible effects of semantic features beyond the face–inanimate object distinction, for both intermediate and target *visual objects*. Although we constrained intermediate fixations to inanimate objects, we cannot rule out the possibility that the effects we observed are modulated by semantic features at this and the target location. For example, text (Cerf et al., 2009; Mackay et al., 2012) or food may be capable of eliciting fast, low-latency saccades as well, and the saccadic “pull” of such features most likely interacts with that of the intermediate fixation target. Future studies could also consider intermediate face fixations and compare face-to-face versus face-to-inanimate object saccades. This may require targeted stimuli and controls, given that scenes with multiple faces tend to come with compositional biases (e.g., faces appearing at the same height). Finally, given the evidence for strong individual traits in gaze behavior (Bargary et al., 2017; Broda & de Haas, 2022a, 2022b; de Haas, Iakovidis, Schwarzkopf, & Gegenfurtner, 2019; Linka, Broda, Alsheimer, de Haas, & Ramon, 2022; Linka & de Haas, 2020; Rigas et al., 2016; Yoon et al., 2020), even larger data sets may allow individual estimates of the effects we found here. Testing the interindividual covariance of latency and velocity effects could provide valuable evidence regarding a core hypothesis suggested by our results: The effect of faces on saccadic latency and velocity may rest on separate mechanisms.

## Conclusion

In summary, we found evidence that faces in complex scenes elicit rapid saccades. Face-directed saccades have higher peak velocity across the amplitude spectrum. This effect is substantial (about 10% of that of the main sequence) and increases across the duration of a trial and for saccades following the trajectory of the preceding saccade. It may reflect mechanisms utilizing memory of previously fixated face locations and/or processes with a wide visual field coverage. Face-directed saccades are also preceded by shorter fixation durations. However, this effect is limited to small saccades early in a trial, which follow the trajectory of the preceding one. This may reflect the perisaccadic shift of an attentional window and face processing mechanisms with a strong parafoveal bias. Thus, the dynamics of natural vision appear to be modulated by several interacting mechanisms, allowing the processing of high-level features outside the fovea.

## Supplementary Material

Supplement 1
