# Supplementary material for: Faces in scenes attract rapid saccades
Source: J Vis. 2023 Aug 8;23(8):11. doi: 10.1167/jov.23.8.11 (PMC10411644; doi:10.1167/jov.23.8.11)
Supplement: Supplement 1 [file jovi-23-8-11_s001.docx]

**Supplementary Information**

# 1 Mixed-effects Models

## 1.1 Model Structure


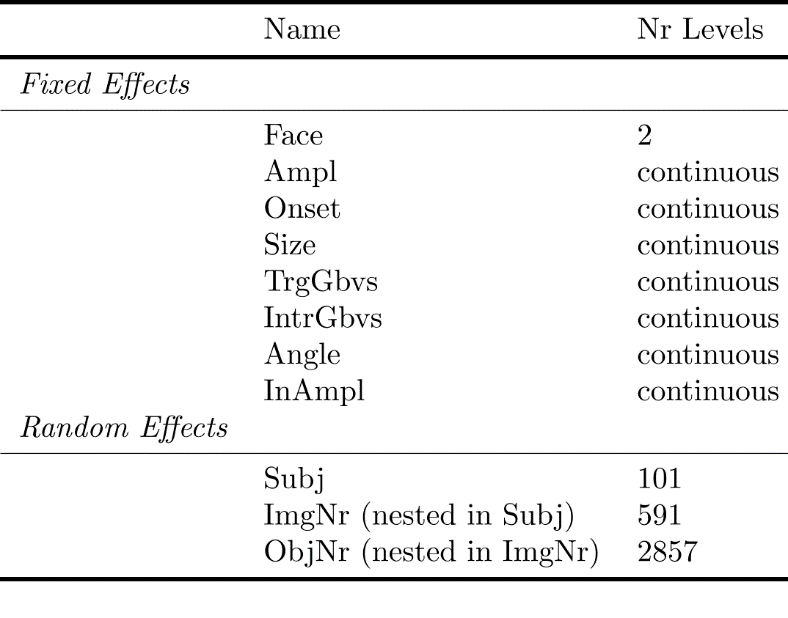


**Table S1.** **Linear mixed-effects models structure for peak velocity and fixation duration**. The table shows the structure of models including eight fixed effects terms: semantic category (Face), target amplitude (Ampl), time from trial onset (Onset), size of target stimuli (Size), low-level salience at target fixation (TrgGbvs), low-level salience at intermediate fixation (IntrGbvs), angle of target amplitude to incoming amplitude (Angle) and the amplitude of incoming saccade (InAmpl). And three random effects terms. subject (Subj) with 101 levels, image (ImgNr) with 591 levels (out of 700 images), and visual object (ObjNr) with 2857 levels (out of 5551 objects). We used dummy coding, with faces and inanimate objects coded as 1 and 0, respectively.

## 1.2 Simple Main Effects


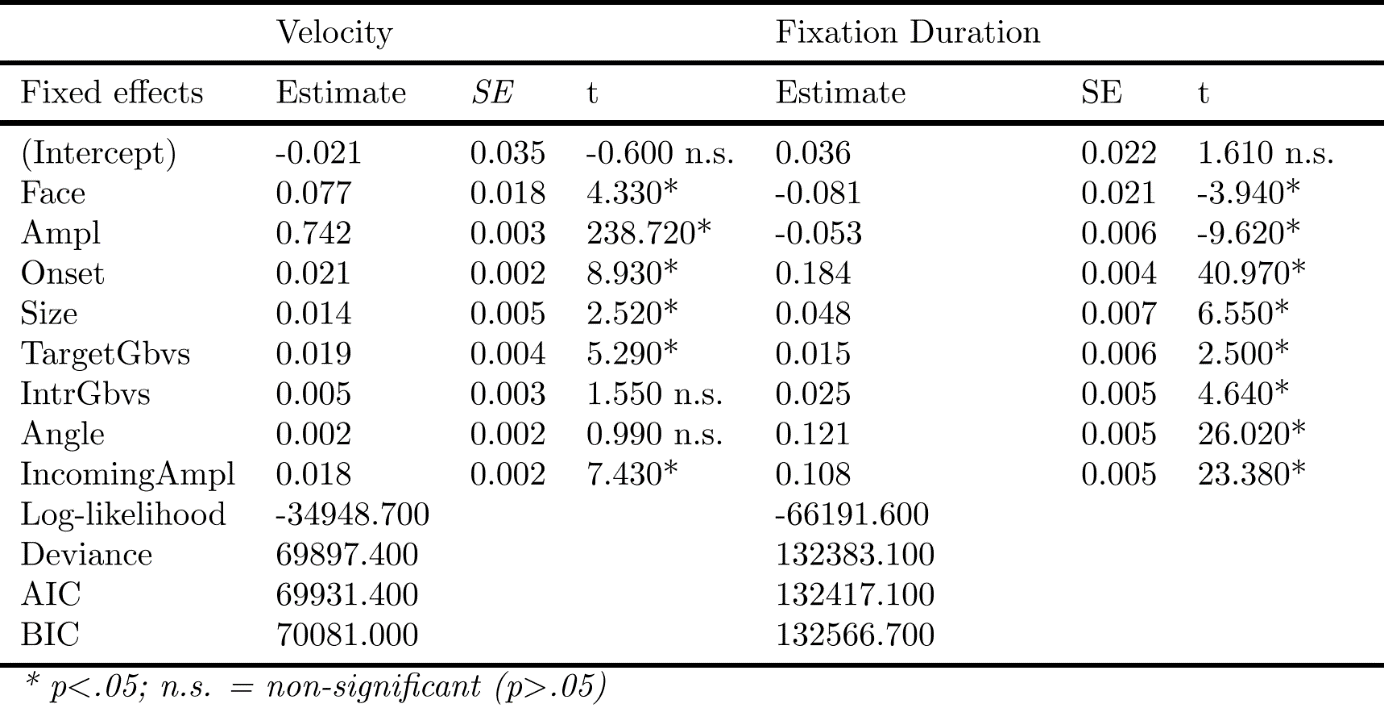


**Table S2**. **Linear mixed-effects models fitting standardized velocity and log fixation duration.** The table shows all simple main effects of the semantic category (Face), target amplitude (Ampl), time from trial onset (Onset), size of target stimuli (Size), low-level salience at target fixation (TrgGbvs), low-level salience at intermediate fixation (IntrGbvs), angle of target amplitude to incoming amplitude (Angle) and the amplitude of incoming saccade (InAmpl). Asterisks in the column of t-values indicate statistically significant beta coefficients. All continuous predictors were z-scored and fixation duration was additionally log-transformed.

## 1.3 Random Effects


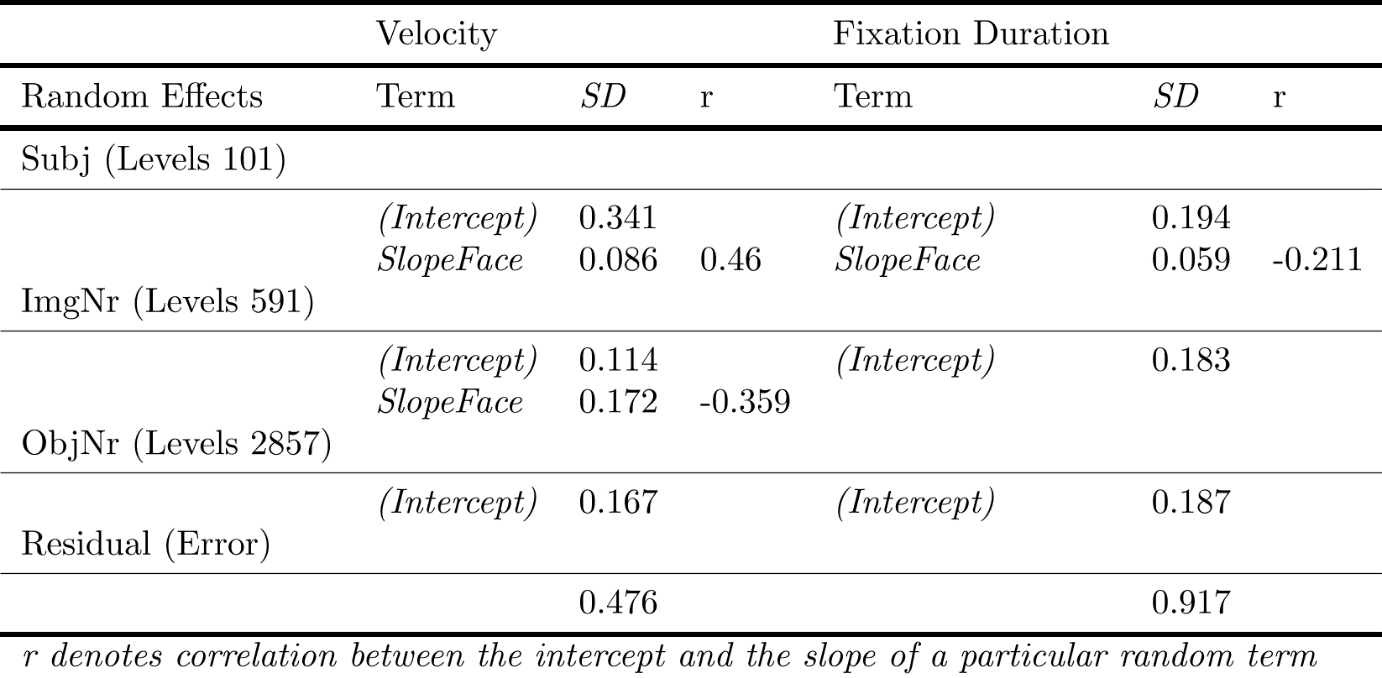


**Table S3**. **Linear mixed-effects models: Estimates of covariance parameters fitting standardized velocity and log fixation duration.** The table shows random effects estimates for random by-subject (Subj) slope and intercept with 101 levels, random by-image (ImgNr) intercept (and slope only for velocity) with 591 levels, and random by-visual object (ObjNr) intercept with 2857 levels. Standard deviations (SD) are reported for each random term and the residual. Random slope for image number was not part of the winning fixation duration model (see AIC comparisons below; Table S7). Correlation coefficients (r) indicate the relationship between intercepts and slopes for a particular random term.

## 1.4 Model Diagnostics: Velocity


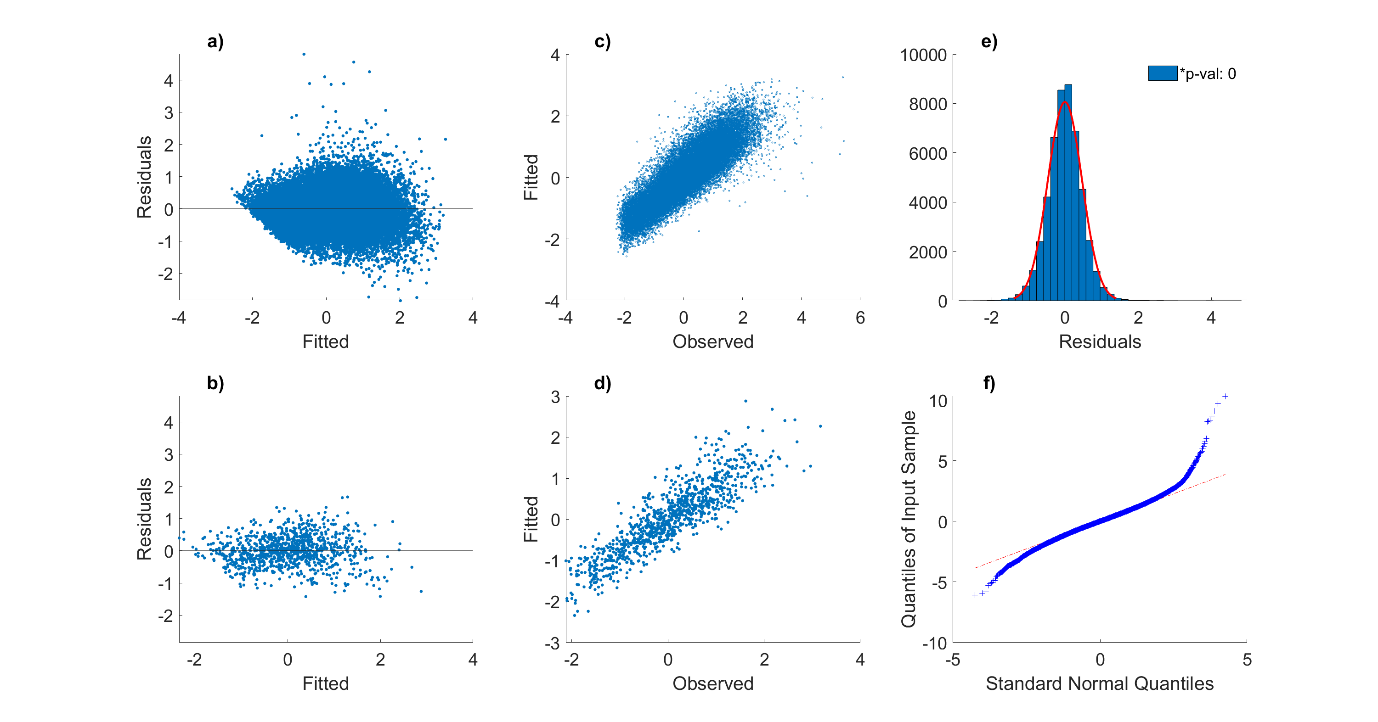


**Figure S1**. **Diagnostic plots of linear mixed-effects model fitting standardized velocity.** Panel (a) shows residuals as a function of fitted values with the full sample (N = 48,881). To alleviate overplotting, a randomly selected subsample of N = 1000 is shown in panel (b). There is no obvious heteroscedasticity or violation of normality. Panel (c) shows fitted values as a function of observed values, with a clear linear relationship. Similarly, panel (d) shows a subsample of fitted vs. observed values (N = 1000). Panel (e) shows a histogram of residuals, approximating a normal distribution, although a non-parametric test indicated a deviation (Kruskal-Wallis Test, *p-val < .001). Panel (f) shows a Q-Q (quantile-quantile) plot, more revealing of normality violations of residuals at the tails of the distribution. We were not able to achieve a better fit by transforming the data.


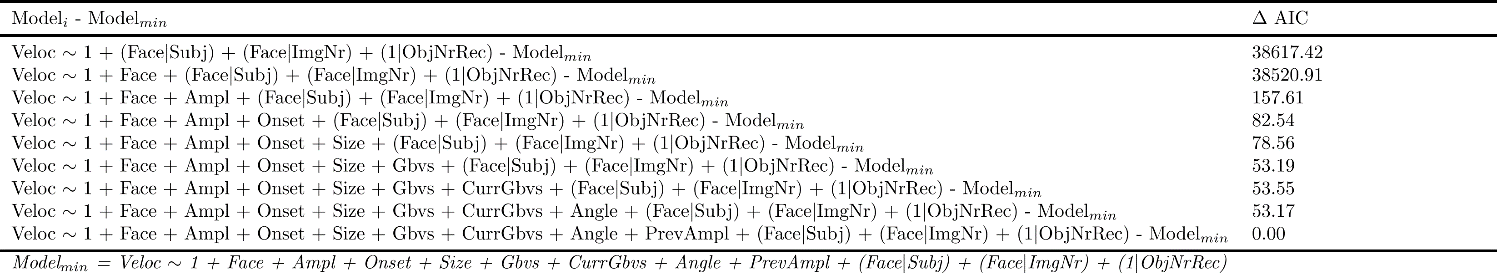


**Table S4.** **AIC comparison of fixed effects for linear mixed-effects models fitting standardized velocity.** The table shows AIC difference values (Δ) for fixed effects comparing model with lowest AIC (Model_min_) to other candidate models (Model_i_). Other candidate models were created by always removing one additional main predictor (Model_i_) and calculating its AIC. As long as the AIC difference (Δ) between minimal and ith model, was no larger than 2, the model with minimal AIC was selected. That was the case for this model of the peak velocity, where the model with minimal AIC was also the most complex model with all respective predictors: Ampl, Onset, Size, Gbvs, CurrGbvs, Angle, and PrevAmpl.


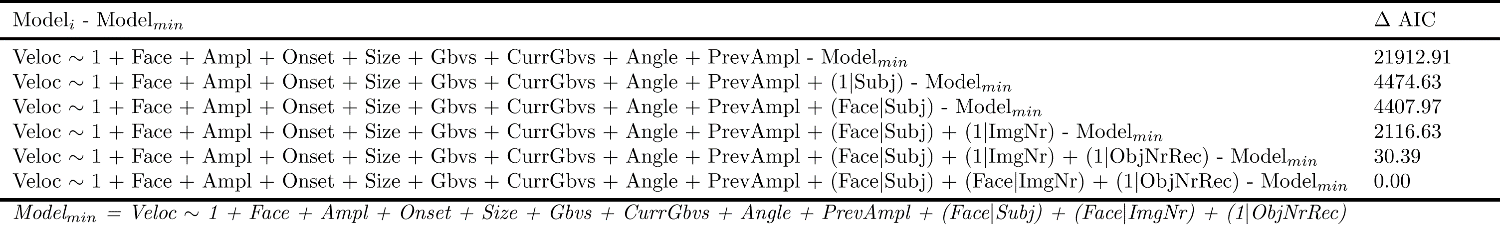


**Table S5.** **AIC comparison of random effects for linear mixed-effects models fitting standardized velocity.** The table shows AIC difference values (Δ) for random effects comparing model with lowest AIC (Model_min_) to other candidate models (Model_i_). Other candidate models were created by always removing one additional random term (Model_i_) and calculating its AIC. As long as the AIC difference (Δ) between minimal and ith model, was no larger than 2, the model with minimal AIC was selected. That was the case for this model of the peak velocity, where the model with minimal AIC was also the most complex model with full random structure: (Face|Subj), (Face|ImgNr), and (1|ObjNrRec).

## 1.5 Model Diagnostics: Fixation Duration


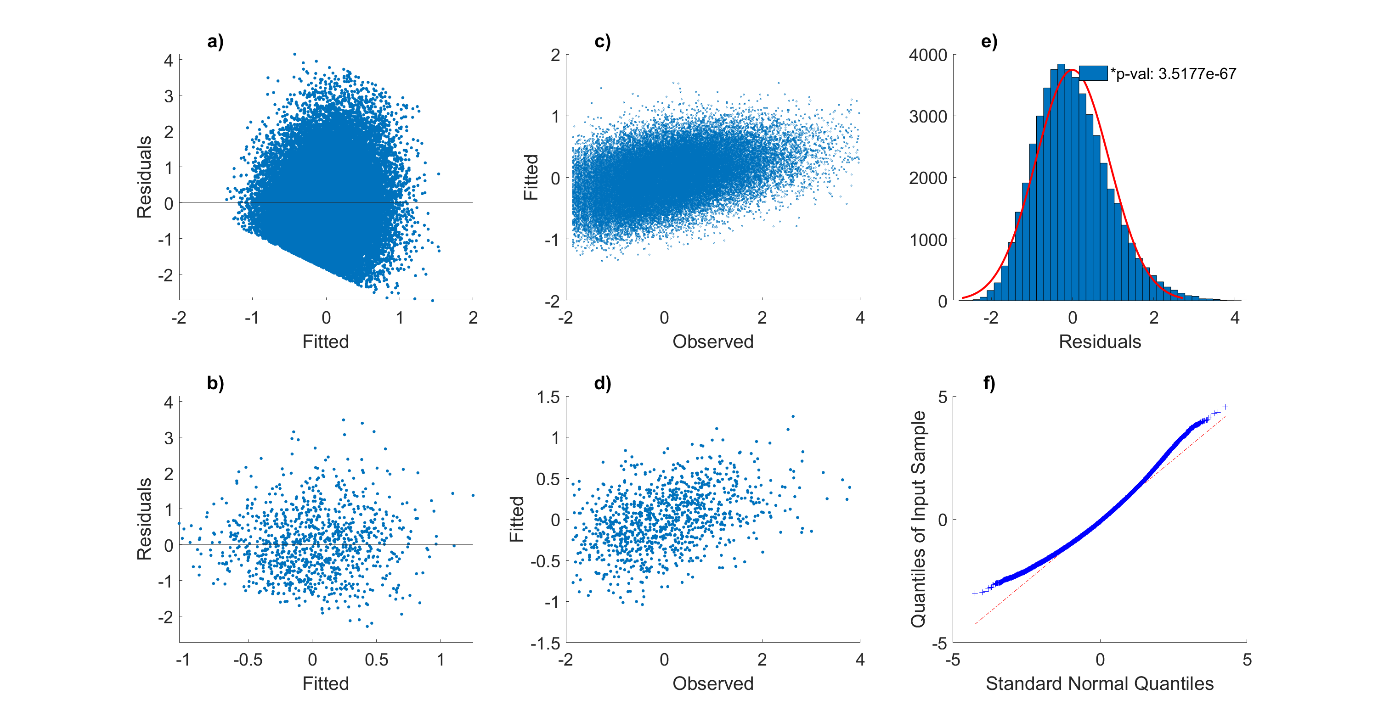


**Figure S2**. **Diagnostic plots of linear mixed-effects model fitting log fixation duration.** Panel (a) shows residuals as a function of fitted values with the full sample (N = 48,881). Due to a large dataset, randomly selected subsample of N = 1000 is shown in panel (b). There seems to be no obvious pattern suggesting heteroscedasticity or violation of normality, although residuals are cluttered narrowly around the center. Panel (c) shows fitted values as a function of observed values, suggesting a linear relationship. Similarly, panel (d) shows a subsample of fitted vs. observed values (N = 1000). Panel (e) shows a histogram of residuals, approximating a normal distribution, although a non-parametric test indicates some deviation from normality (Kruskal-Wallis Test, *p-val < .001). Panel (f) shows a Q-Q (quantile-quantile) plot revealing minor deviations from normality at the tails.


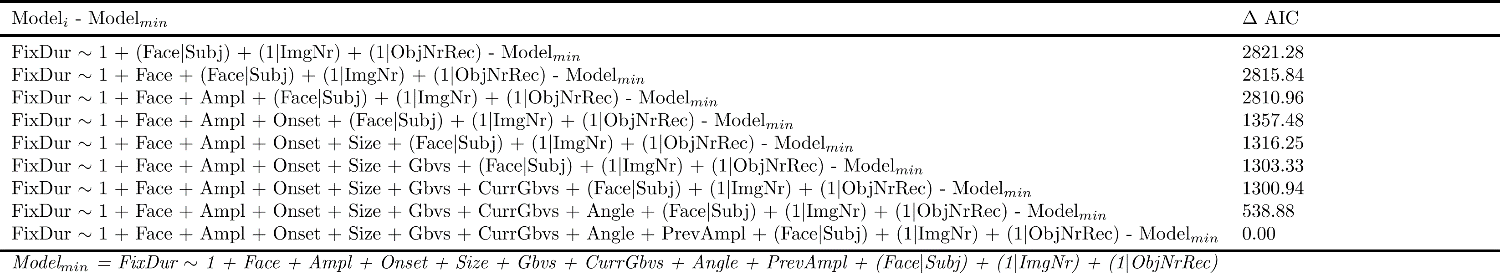


**Table S6**. **AIC comparison of fixed effects for linear mixed-effects models fitting log fixation duration.** The table shows AIC difference values (Δ) for fixed effects comparing model with lowest AIC (Model_min_) to other candidate models (Model_i_). Other candidate models were created by always removing one additional main predictor (Model_i_) and calculating its AIC. As long as the AIC difference (Δ) between minimal and ith model, was no larger than 2, the model with minimal AIC was selected. That was the case for this model of the fixation duration, where the model with minimal AIC was also the most complex model with all respective predictors: Ampl, Onset, Size, Gbvs, CurrGbvs, Angle, and PrevAmpl.


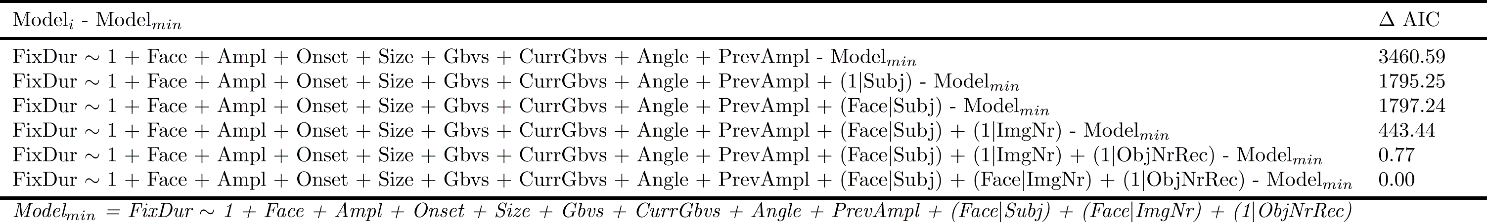


**Table S7.** **AIC comparison of random effects for linear mixed-effects models fitting standardized velocity.** The table shows AIC difference values (Δ) for random effects comparing model with lowest AIC (Model_min_) to other candidate models (Model_i_). Other candidate models were created by always removing one additional random term (Model_i_) and calculating its AIC. As long as the AIC difference (Δ) between minimal and ith model, was no larger than 2, the model with minimal AIC was selected. This was not the case for this model of fixation duration, as the more simple model had AIC difference lower than 2. Therefore, simpler model of the fixation duration was selected with following random structure: (Face|Subj), (1|ImgNr), and (1|ObjNrRec).

# 2 Anova


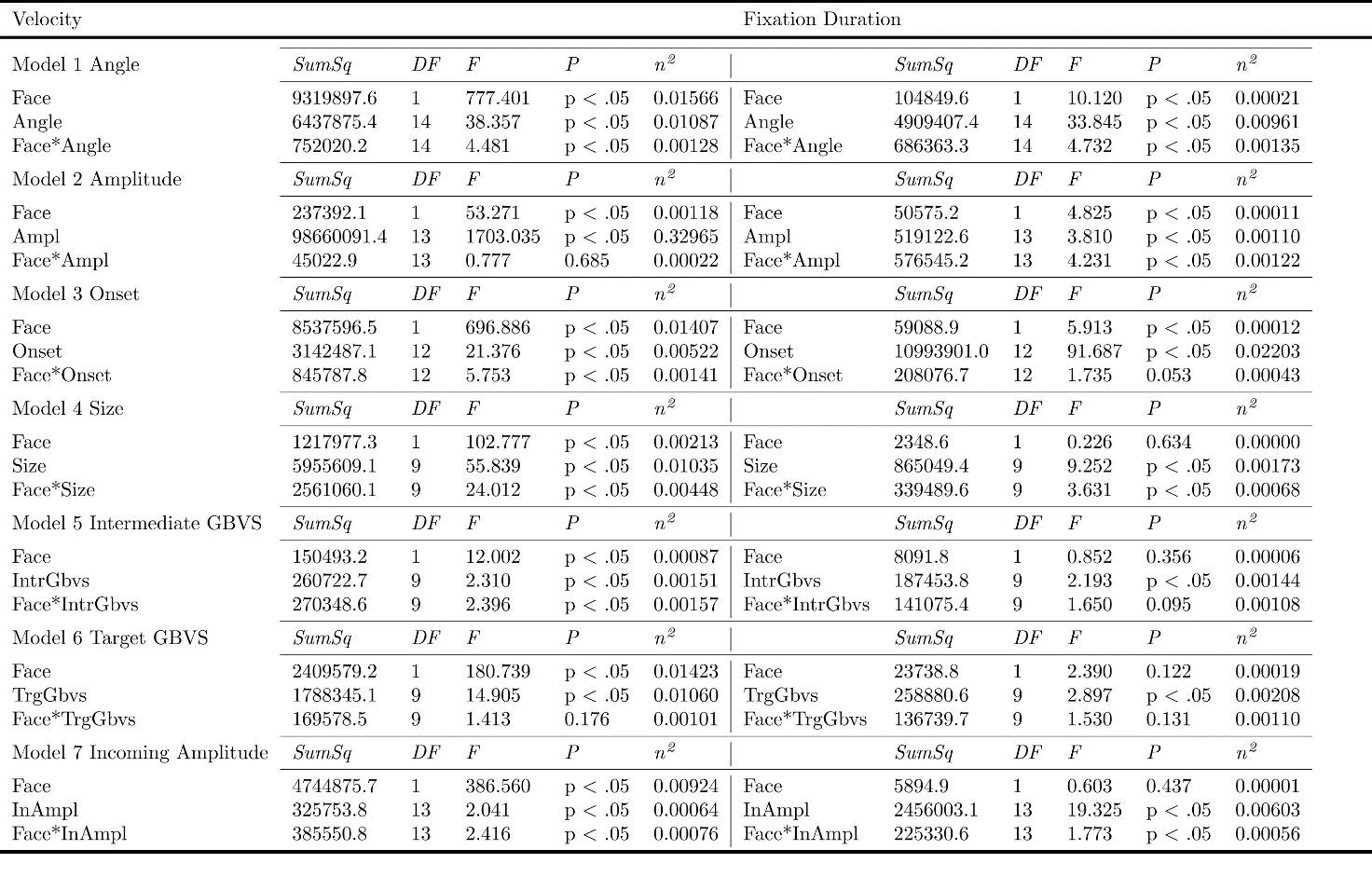


**Table S8**. **Two-way ANOVAs for peak velocity and fixation duration**. Each model tested simple main effects and an interaction of semantic target category with one of the following predictors: absolute deviation of saccade angles between target and incoming saccade (Model 1); target saccade amplitude (Model 2); time from trial onset (Model 3); size of the target stimuli (Model 4)); low-level salience at intermediate fixation (Model 5); low-level salience at target fixation (Model 6); amplitude of the incoming saccade (Model 7).

# 3 Remaining predictors


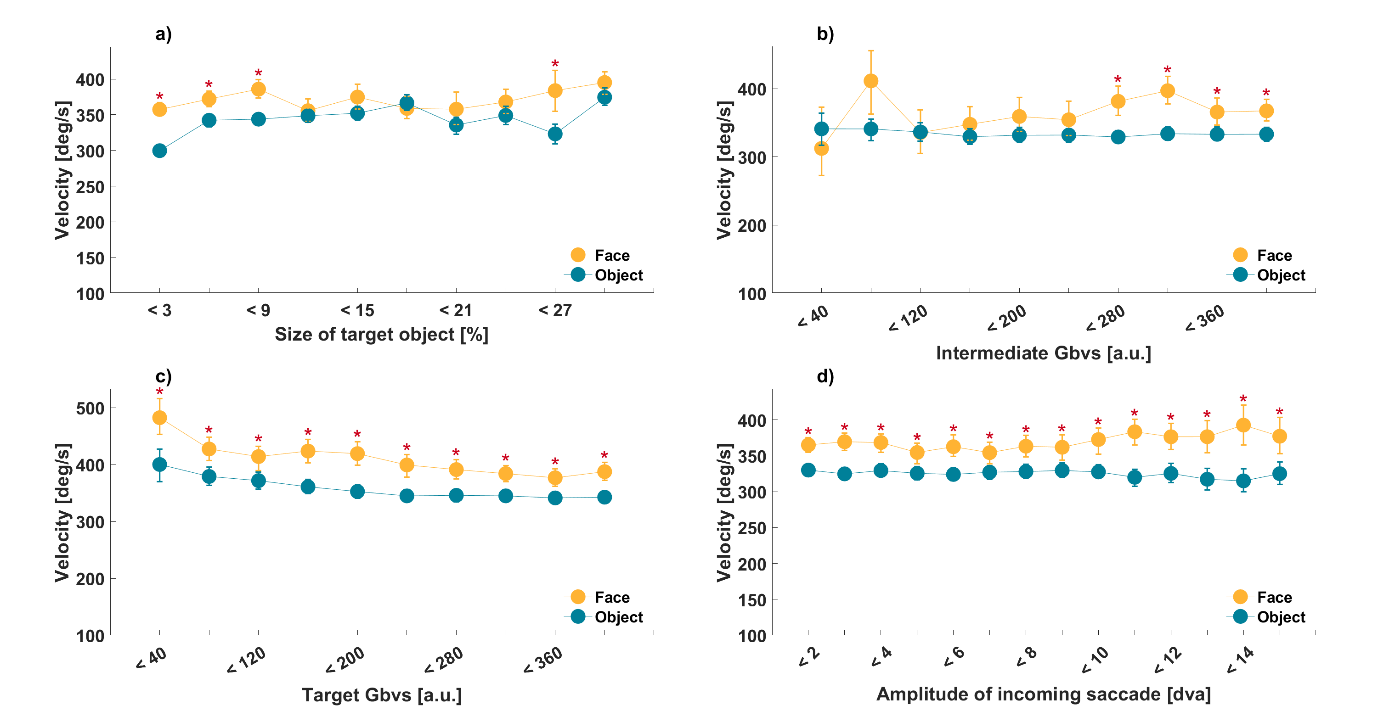


**Figure S3.** **Differences in peak velocity between face- vs. inanimate object-directed saccades.** Red asterisks mark Bonferroni corrected significance of paired t-test and error bars represent bootstrapped 95 % confidence interval (1,000 resamples). Panel (a) shows peak velocity as a function of size of target stimuli (Size). Panel (b) shows peak velocity as a function of low-level salience at the intermediate fixation. Panel (c) shows peak velocity as a function of low-level salience at the target fixation. Panel (d) shows peak velocity as a function of amplitude of the incoming saccade. Low-level salience is given in arbitrary units (a.u.); low values indicate low saliency and high values indicate high saliency. Cyan and yellow markers denote data from inanimate object and face-directed saccades as shown in the inset.


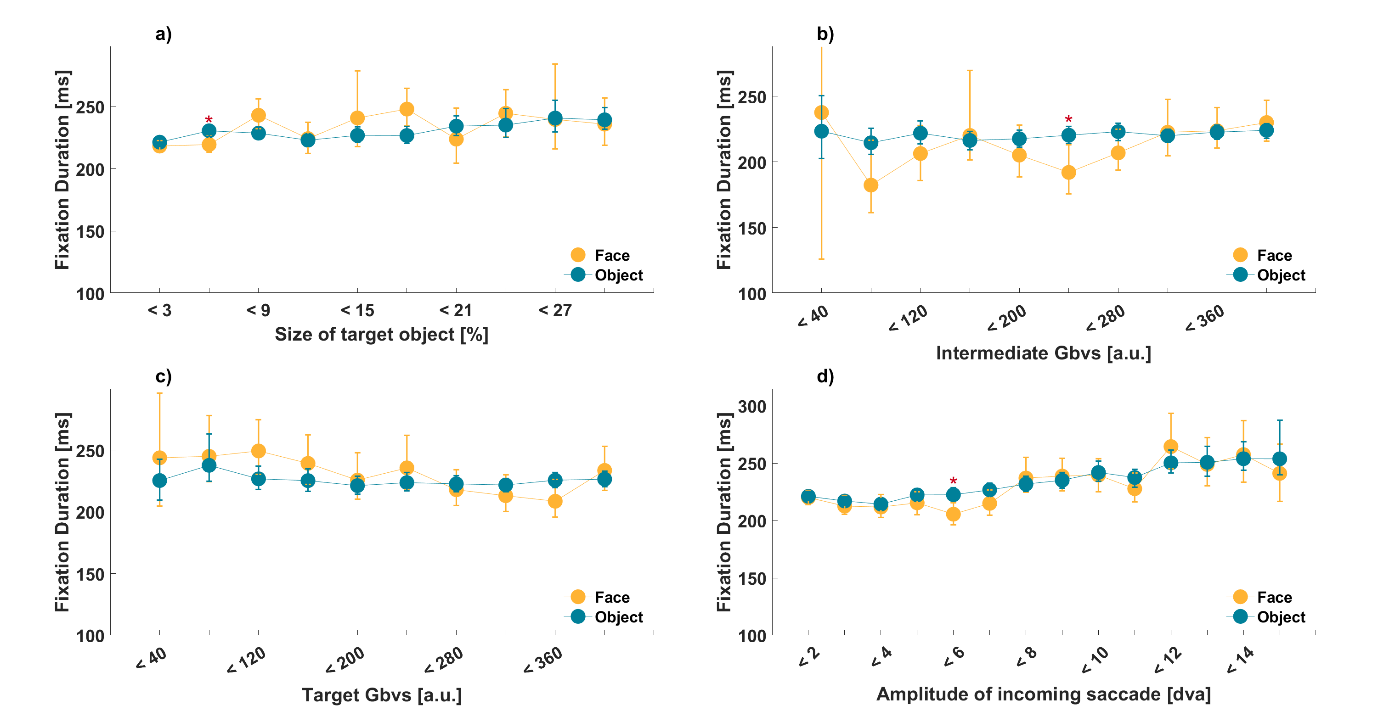


**Figure S4**.  **Differences between intermediate fixation durations preceding face- vs. inanimate object-directed saccades.** Red asterisks mark Bonferroni corrected significance of paired t-test and error bars represent bootstrapped 95 % confidence interval (1,000 resamples). Panel (a) shows fixation duration as a function of size of target stimuli (Size). Panel (b) shows fixation duration as a function of low-level salience at the intermediate fixation. Panel (c) shows fixation duration as a function of low-level salience at the target fixation. Panel (d) shows fixation duration as a function of amplitude of the incoming saccade. Low-level salience is given in arbitrary units (a.u.); low values indicate low saliency and high values indicate high saliency. Cyan and yellow markers denote data from inanimate object and face-directed saccades as shown in the inset.

# 4 Control Analysis: Amplitude


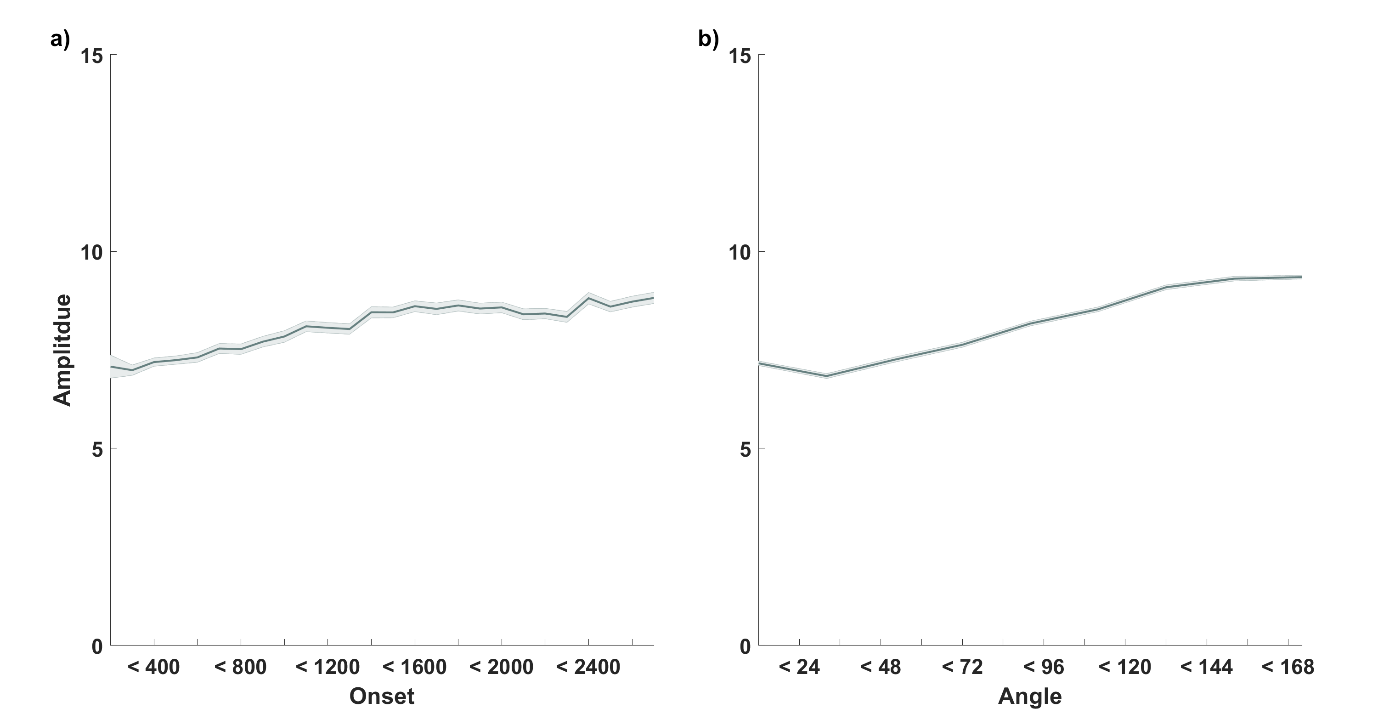


**Figure S5**. **Target amplitude as a function of trial time and relative saccadic angle**. Panel (a) shows the amplitude of target saccades as a function of the time across trial duration. Mean amplitudes were calculated using a sliding window with a width of 100 ms. Panel (b) shows target amplitude as a function of the angle between incoming and target saccade. Mean amplitudes were calculated using a sliding window with a width of 40 deg. Shaded areas represent the standard error of the mean.


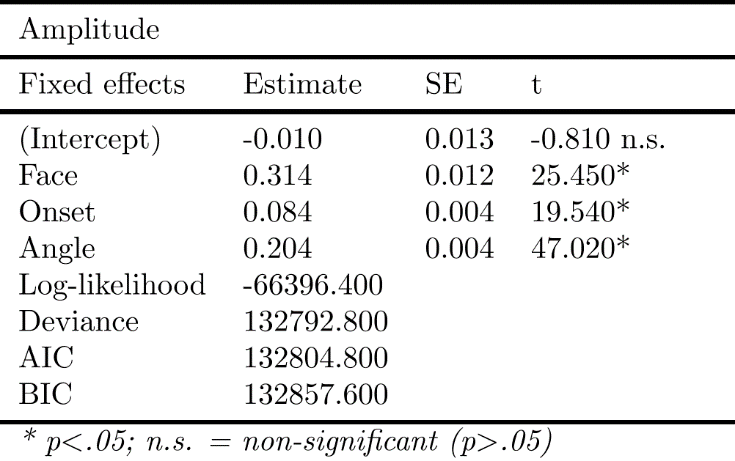


**Table S9.** **Linear mixed-effects models fitting standardized amplitude.** The table shows simple main effects of sematic category (Face), time from trial onset (Onset), and angle of target amplitude to incoming amplitude (Angle). Asterisks in the column of t-values indicate statistically significant beta coefficients. All continuous predictors were z-scored.

# 5 Target Size


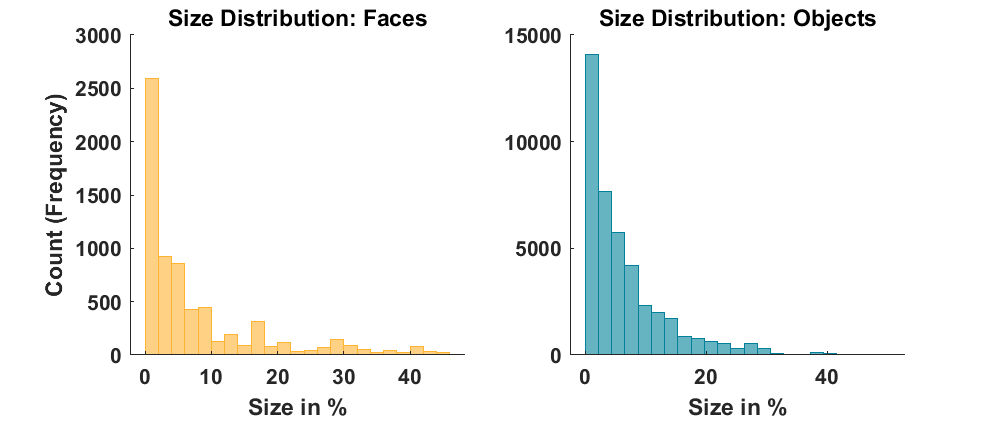


**Figure S6. Distribution of the size of inner faces and inanimate objects**. The figure shows the number of inner face masks and inanimate object masks by their size expressed as % of the total image size. Images size was 29.7 x 22.3 degrees visual angle and the majority of faces was not larger than 3 dva.

# 6 Animacy in Images


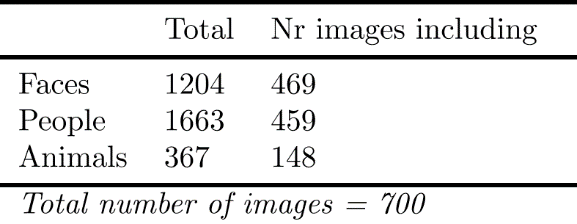


**Table S10. Frequency of faces, people, and animals in the images**. The total number of images was 700, out of that 469 contained faces, 459 contained people, and 148 contained animals. No intermediate or target fixations falling on animals were included.
